# Synthesis of Lignin-Derived Hierarchical Porous Carbon via Hydrothermal–Phosphoric Acid Synergistic Activation for Enhanced Adsorption of Tetracycline

**DOI:** 10.3390/molecules31030447

**Published:** 2026-01-27

**Authors:** Xin Li, Yipeng Li, Yuhan Li, Mengyu Zhang, Jundong Zhu

**Affiliations:** 1 Hunan Provincial Key Laboratory of the Research and Development of Novel Pharmaceutical Preparations, College of Pharmacy, Changsha Medical University, Changsha 410219, China; 2 Hunan Provincial Key Laboratory of Carbon Neutrality and Intelligent Energy, School of Resources and Environment, Hunan University of Technology and Business, Changsha 410205, China

**Keywords:** lignin, mesoporous structure, carbon, tetracycline, adsorption

## Abstract

Tetracycline is a low-cost broad-spectrum antibiotic and widely used in medicine and aquaculture. Its residues are usually released into the environment through wastewater, which may lead to the spread of antibiotic resistance genes and pose ecological risks. To address this environmental issue, a hierarchical lignin-derived porous carbon (LPHC) was synthesized using renewable biomass lignin as the precursor through a combined phosphoric acid-activated hydrothermal pretreatment. The resulting LPHC was used to effectively remove tetracycline from aqueous solutions. Characterization results indicated that LPHC had a high specific surface area (1157.25 m^2^·g^−1^), a well-developed micro-mesoporous structure, and abundant surface oxygen-containing functional groups, which enhanced its interaction with target pollutants. Adsorption experiments showed that LPHC exhibited excellent adsorption performance for tetracycline, with a maximum adsorption capacity of 219.81 mg·g^−1^. The adsorption process conformed to the Langmuir isotherm model, indicating that monolayer chemical adsorption was dominant. Mechanism analysis further confirmed that the adsorption process was controlled by multiple synergistic interactions, including pore filling, π-π electron donor–acceptor interactions, hydrogen bonding, and electrostatic attraction. This work proposes a feasible strategy to convert waste biomass into high-performance and environmentally friendly adsorbents, which provides technical feasibility for sustainable water purification technologies.

## 1. Introduction

Antibiotics are chemical compounds that inhibit or eliminate pathogenic microorganisms by disrupting essential cellular processes and are extensively employed in human medicine, animal husbandry and aquaculture [1,2,3,4]. However, the widespread and frequently indiscriminate global use of antibiotics has resulted in the continuous discharge of these drugs into aquatic systems via pathways such as medical effluent, agricultural runoff and livestock waste [4,5]. Consequently, antibiotics have become persistent and ubiquitous contaminants in water bodies. These residues not only present potential risks to human health through bioaccumulation and food-chain transfer but also foster the emergence and spread of antimicrobial resistance, compounding a serious worldwide public health issue [6,7,8]. Conventional water treatment methods, which predominantly depended on physical sedimentation, biological degradation, and conventional disinfection, generally exhibited limited efficacy in removing antibiotic residues [9,10]. Therefore, there is a pressing need to develop efficient and environmentally sustainable advanced technologies to mitigate antibiotic pollution.

Currently, common strategies for removing antibiotics from water include degradation, biodegradation, and adsorption [11,12,13,14,15,16]. Among these, adsorption has garnered significant attention owing to its high removal efficiency, operational simplicity, and cost-effectiveness [17,18,19]. The performance of adsorption largely depends on the selection of adsorbent material, with commonly studied options including graphene, metal–organic frameworks, activated carbon, mineral-based adsorbents, and biochar [20,21,22]. Biochar is particularly notable for its tunable pore structure, diverse surface functionalities, and economic production [23]. It can be produced from abundant biomass resources such as agricultural and forestry residues, offering distinct advantages in sustainability and environmental compatibility [24,25]. The adsorption capacity of biochar is closely linked to its textural properties, which can be strategically modulated to enhance its affinity toward antibiotics [26]. With the increasing efforts to control water pollution, biomass-derived porous carbon materials show great potential in treating water contaminated with antibiotics.

Lignin, the most abundant renewable aromatic polymer in nature, possesses abundant active sites such as phenolic hydroxyl and methoxy groups [27,28]. As a major byproduct of the pulp and paper industry, it has long been regarded as low value waste and currently has a utilization rate of less than 5% [29]. Some studies have shown that lignin’s aromatic skeleton make it an ideal precursor for preparing high-performance porous carbon materials [30,31]. Moreover, porous carbons with hierarchical pore structures can be prepared by using activators such as phosphoric acid, zinc chloride, hydroxides, and carbonates. The resulting materials have high specific surface area and porosity and exhibit good adsorption performance [18,32,33]. Nevertheless, single activation methods often face challenges such as uneven impregnation and incomplete pore development, which limit the effective diffusion of macromolecular antibiotics and result in relatively low adsorption capacities for tetracycline antibiotics. Moreover, different activators have their own limitations in the pore-forming process. Zinc chloride is prone to causing pore collapse and blockage; strong bases like potassium hydroxide or sodium hydroxide, although having strong etching capabilities, are likely to cause excessive corrosion and form structures dominated by micropores; while phosphoric acid, although helpful in promoting pore generation, is sensitive to process conditions and may affect the uniformity of the final pore size distribution [33,34]. To address these issues, Zhou et al. used hydrothermal carbonization to convert lignin into aromatic carbon materials rich in oxygen-containing functional groups, and then combined KOH activation to further increase the porosity and defect density, ultimately obtaining a highly efficient antibiotic adsorbent with a honeycomb-like structure [35]. Samba et al. prepared porous carbon from cannabis residue via phosphoric acid-assisted hydrothermal carbonization followed by thermal treatment. Compared to conventional phosphoric acid activation, the introduced hydrothermal step effectively tailored the pore structure, exhibiting enhanced adsorption toward antibiotics [36]. Therefore, the adsorbents prepared through the directional regulation of the porous structure of lignin-based carbon materials show broad application prospects in the efficient removal of antibiotics.

Herein, a hierarchical porous carbon material derived from lignin was successfully synthesized via phosphoric acid-assisted hydrothermal pretreatment coupled with pyrolysis activation. This two-step synergistic mechanism proceeds as follows: firstly, during the phosphoric acid hydrothermal pre-carbonization stage, phosphoric acid molecules penetrate deeply into the lignin matrix, promoting the directional depolymerization of macromolecular segments. Subsequently, in the programmed pyrolysis stage, phosphoric acid acts as a bifunctional agent, not only facilitating the formation of a carbonaceous precursor but also serving as a pore-forming agent to regulate the evolution of porous structures. Compared with porous carbons prepared by conventional direct pyrolysis or simple phosphoric acid impregnation–carbonization, the resulting hierarchical porous carbon exhibits a well-developed pore network, retains abundant oxygen-containing functional groups, and demonstrates enhanced adsorption capacity for tetracycline. This structure–function synergy regulation strategy offers a promising route for developing high-performance lignin-derived carbon materials to address antibiotic contamination in wastewater treatment systems.

## 2. Results

### 2.1. Morphology Characterization

**Figure 1:**
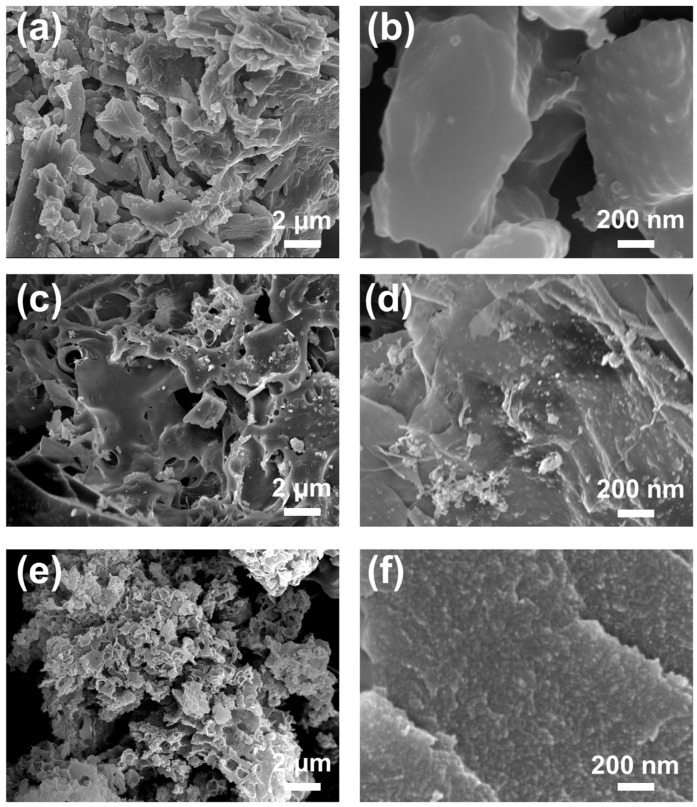
SEM images and corresponding large-scale SEM images: (**a**,**b**) LC, (**c**,**d**) LPC and (**e**,**f**) LPHC.

Figure 1 illustrates the morphological differences among lignin-derived carbon materials prepared via distinct carbonization processes. The LC exhibits a dense, block-like macro-morphology with minimal visible porosity (Figure 1a). The corresponding high-magnification image (Figure 1b) reveals a surface characterized by localized collapse features and disordered stacking of carbon layers. For LPC (Figure 1c), the introduction of phosphoric acid activation can promote the formation of a porous morphology in the carbon material. These pores were surrounded by relatively thick pore walls and had limited connectivity (Figure 1d). This structural feature indicates that although phosphoric acid functioned as a dehydrating agent and crosslinking agent during the reaction, the single-step activation process led to incomplete pore development and insufficient construction of a continuous pore network. In contrast, LPHC (Figure 1e) exhibits a three-dimensional porous structure, with its structure composed of a large number of interpenetrating pores, forming a distinct hierarchical network. Additionally, its surface shows a distinct rough texture (Figure 1f), indicating continuous etching during the synergetic hydrothermal and pyrolysis process. This hydrothermal pretreatment promoted the homogenization and pre-porosity of the precursor, and the subsequent phosphoric acid-guided pyrolysis process successfully constructed an open multi-level pore structure while stabilizing the carbon skeleton. These results indicate that the pore characteristics of the material were mainly regulated by the activation strategy. Compared with direct carbonization and simple phosphoric acid activation, the hydrothermal–phosphoric acid synergistic process can promote the structural reorganization of the precursor, thereby stabilizing the carbon skeleton while constructing a multi-level pore structure.

### 2.2. Structure and Chemical Characterization

**Figure 2:**
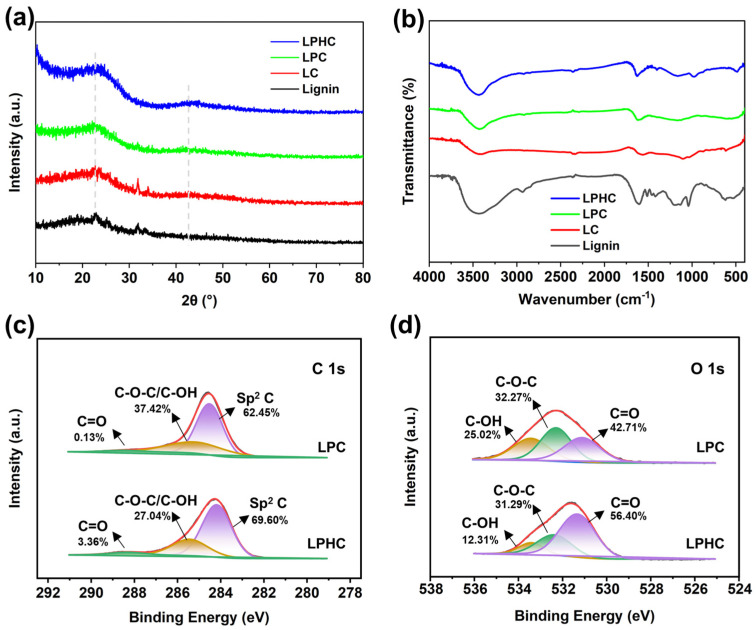
(**a**) XRD patterns of lignin and three derived carbons. (**b**) FTIR spectra of different adsorbents and lignin. The high-resolution XPS spectra of (**c**) C 1s and (**d**) O 1s of LPHC and LPC.

Figure 2a shows the XRD patterns of the original lignin and three derived carbon materials. The resulting overall carbon materials all exhibit the crystal structure of amorphous carbon sheets. The pattern for LC exhibited a broadened (002) diffraction peak at approximately 2θ ≈ 22°, characteristic of typical amorphous carbon with disordered stacking of carbon layers [37]. The low-intensity peaks originate from the aromatic structural fragments that have not been completely pyrolyzed [38], which were close to the diffraction peaks of the original lignin. The LPC exhibits two distinct and broadened diffraction peaks at approximately 25° and 43°, which were, respectively, attributed to the (002) and (100) crystal planes of graphite carbon [39]. Notably, LPHC exhibited a significantly enhanced (002) diffraction peak, which is attributed to the synergistic effect of phosphoric acid activation after hydrothermal pretreatment. Hydrothermal phosphoric acid treatment induces reorganization and partial pre-graphitization of the lignin precursor, while subsequent pyrolysis promoted the growth and ordered stacking of graphitic microdomains within the resulting structure. Figure 2b compares the FTIR spectra of lignin and its derived carbon materials. The FTIR spectrum of pine wood lignin showed typical characteristic peaks at 3420 cm^−1^ (O–H), 2930 cm^−1^ (C–H), 1600 cm^−1^ (aromatic C=C), and 1270 cm^−1^ (C–O–C) [31,40,41]. For LC, the FTIR spectrum showed a significant weakening of oxygen-containing groups (O–H, C–O–C), while the peaks at 1580 cm^−1^ and 613 cm^−1^ are attributed to the C=C stretching vibration and bending vibration of the aromatic ring skeleton, respectively [42], indicating the formation of a low-substituted aromatic structure. For LPC and LHPC prepared by phosphoric acid activation, the peaks at 3200 cm^−1^ (O–H vibration) and 1580 cm^−1^ (C=C vibration) were still visible, and the peak at 1150 cm^−1^ corresponds to the C–O stretching in the aromatic structure [43], indicating that the carbon skeleton remains stable after activation. Notably, LPHC showed a splitting of the C=C and C=O vibration peaks at 1580 cm^−1^ and 1320 cm^−1^ [44]. This change may be related to the rearrangement of graphite layer stacking and the increase in surface oxygen-containing functional groups caused by the phosphoric acid activation process. The above results indicate that different physical methods of phosphoric acid activation can effectively regulate the surface chemical properties of carbon materials.

To further clarify the surface elemental composition and chemical states of LPHC, XPS analysis was conducted. As shown in Figure S1, the full spectra of LPC and LPHC confirmed that the surface was mainly composed of carbon, oxygen, and weak nitrogen elements. The high-resolution C 1s spectrum (Figure 2c) could be fitted into three characteristic peaks at 284.2 eV, 285.5 eV, and 288.4 eV, corresponding to sp^2^ carbon (C-C/C=C), C-O bond and carbonyl/carboxyl carbon (C=O) [7,45,46], indicating that carbon existed in the form of aromatic skeletons and various oxygen-containing functional groups. The corresponding O 1s spectrum (Figure 2d) could be deconvoluted into three components of C=O (531.3 eV), C-O-C (532.4 eV), and C-OH (533.4 eV) [29,47], which directly confirmed the existence of abundant and diverse carbon-oxygen bonds on the material surface. Furthermore, the C 1s spectrum of LPHC showed that the C=O content (3.36%) was higher than that of LPC (0.13%), and its sp^2^ C-C and C-O-C/C-OH structures remained stable, indicating that the phosphoric acid hydrothermal process promoted carbon skeleton stability while introducing carbonyl groups. The O 1s spectrum further confirmed that the carboxyl group content of LPHC (derived from C=O) was about 13.7% higher than that of LPC, indicating that the phosphoric acid hydrothermal treatment effectively enhanced the surface acidic oxygen-containing functional groups [7]. In summary, phosphoric acid hydrothermal pretreatment can selectively regulate the skeletal oxidation state and surface functional group distribution of porous carbon.

### 2.3. Specific Surface Area and Pore Size Structure

**Figure 3:**
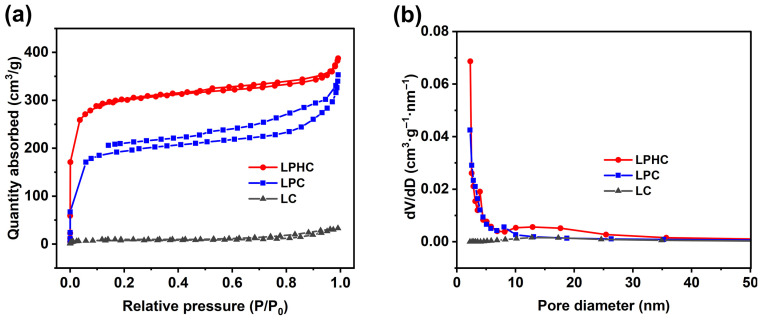
(**a**) Nitrogen adsorption–desorption isotherms and (**b**) pore size distributions of LC, LPC and LPHC.

Figure 3 shows the nitrogen adsorption–desorption isotherms and pore size distribution curves of three carbon materials and summarizes the corresponding physical parameters of the pore structure (see Table 1). The isotherms of LPHC and LPC both exhibited type IV characteristics and have obvious H4-type hysteresis loops [36], indicating the presence of abundant mesoporous structures in the materials; while the isotherm of LC did not show significant hysteresis loops, suggesting that its pore structure development is relatively low. Among them, LPHC had the highest specific surface area (1157.25 m^2^/g) and total pore volume (0.60 cm^3^/g), with an average pore diameter of approximately 2.1 nm. The above results indicated that the synergistic treatment of hydrothermal and phosphoric acid could more effectively regulate the pore structure of the carbon skeleton and achieve directional optimization of the pore structure. The synergistic effect of high specific surface area and hierarchical porous structure is a key structural factor for significantly improving the adsorption performance of the material.

### 2.4. The Adsorption Effect of TC by Lignin-Derived Carbon

**Figure 4:**
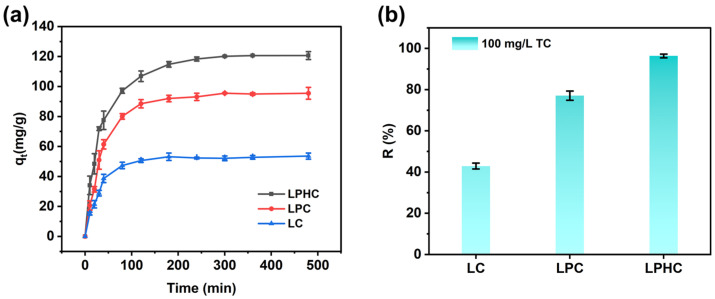
(**a**) Adsorption equilibrium curves of TCs and (**b**) the corresponding removal efficiencies on the LC, LPC and LPHC.

Figure 4 illustrates the variation in adsorption performance ofTC by lignin-derived porous carbon materials synthesized through different methods over time, as well as their equilibrium adsorption capacities and removal efficiencies. As shown in Figure 4a, the adsorption processes of all materials exhibited typical kinetic characteristics. In the initial stage (0–40 min), the adsorption capacity rises rapidly, mainly due to the abundant adsorption sites and the relatively high TC concentration. Subsequently, the rate gradually slows down from 40 to 480 min, indicating that the available adsorption sites were gradually decreasing, and the adsorption equilibrium was ultimately reached around 480 min. Among different materials, the direct carbonized sample LC has the lowest equilibrium adsorption capacity, approximately 54.25 mg/g. The adsorption capacity of LPC, which was activated by phosphoric acid impregnation, increased to 96.33 mg/g, while LPHC, prepared through hydrothermal coupling activation, demonstrated the best adsorption performance, with an equilibrium adsorption capacity as high as 120.63 mg/g and a significantly accelerated adsorption rate. From the corresponding removal efficiency (Figure 4b), LPHC also achieved the highest removal rate, reaching about 96.33%. These results indicated that the hierarchical porous structure constructed by hydrothermal pretreatment effectively optimized the mass transfer channels while maintaining a high specific surface area, thereby synergistically enhancing the adsorption capacity and removal efficiency of the material.

### 2.5. Effects of Solution pH, Adsorbent Dosage, and Initial Substrate Concentration on TC Adsorption by LPHC

**Figure 5:**
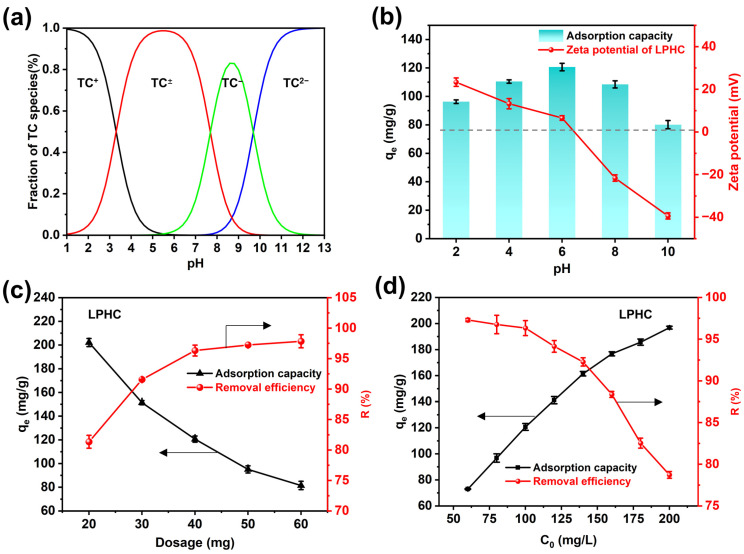
(**a**) pH-dependent species of TC. (**b**) Effects of solution pH on the adsorption of TC by LPHC and zeta potential of LPHC. Effect of (**c**) adsorbent dose and (**d**) Initial concentration of TC on the adsorption of TC by LPHC.

The adsorption performance of LPHC for TC was significantly affected by the solution environment and adsorption conditions. As shown in Figure 5a, based on the pKa value of tetracycline, it can present three forms under different pH conditions: cationic TC^+^ (pH < 3.3), neutral molecular TC^0^ (3.3 < pH < 7.7), and anionic TC^−^/TC^2−^ (pH > 7.7) [48]. Figure 5b shows the trends of the ζ potential and adsorption capacity of LPHC with the change in solution pH. When pH < 3.3, the adsorbent surface was positively charged, generating electrostatic repulsion with TC mainly in the form of TC^+^ [17], which resulted in weak adsorption. As pH increased to 3.3–7.7, TC gradually transformed into electrically neutral TC^0^ [49], reducing electrostatic repulsion and increasing the adsorption capacity. At pH = 6, close to the zero charge point of LPHC (pH_ZPC_ = 6.47), the adsorption capacity reached a maximum of 120.63 mg/g. When pH > 9.7, TC was completely deprotonated to TC^2−^, the negative charge on the adsorbent surface intensifies, electrostatic repulsion increases, and OH^−^ competed for adsorption sites with TC [20], leading to a decrease in adsorption capacity. As shown in Figure 5c, the TC removal rate increased from 81.35% to 96.32% as the dosage increased from 0.02 g to 0.04 g, while the equilibrium adsorption capacity decreased from 202.13 mg·g^−1^ to 120.63 mg·g^−1^. Further increasing the dosage to 0.06 g does not significantly change the removal efficiency. This indicated that increasing the dosage enhanced the overall removal efficiency but reduced the utilization rate of active sites per unit mass of the adsorbent. The effect of the initial TC concentration is illustrated in Figure 5d. Within the range of 60–100 mg·L^−1^, the removal rate remained above 90%; when the concentration rose to 200 mg·L^−1^, the removal rate dropped to 78.72%, and qₑ increased linearly with the initial concentration, suggesting that at lower concentrations, there were sufficient active sites, but at higher concentrations, they tend to become saturated. The solution pH played a dominant role in adsorption by regulating the surface charge of the adsorbent and the form of TC. Therefore, electrostatic interaction has a key influence on the adsorption process, and optimizing the adsorption process of LPHC requires a balance between enhancing removal efficiency and maintaining high adsorption capacity.

### 2.6. Study on the Adsorption Performance of LPHC for TC

**Figure 6:**
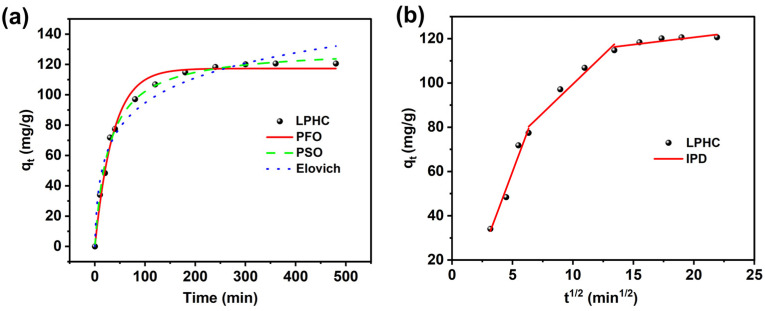
(**a**) Pseudo-first-order and pseudo-second-order kinetics model plots; (**b**) intraparticle diffusion model plots. (Adsorption conditions: *C_0_* = 100 mg/L; adsorbent dose = 0.04 g; *V_0_* = 50 mL, pH = 6).

As presented in Table 2, The adsorption kinetics mechanism of tetracycline on LPHC was investigated by fitting the experimental data with pseudo-first-order, pseudo-second-order and Elovich models (Figure 6a). As shown in Table 2, the correlation coefficients of the pseudo-second-order (*R^2^* = 0.995), pseudo-first-order (*R^2^* = 0.987) and Elovich (*R^2^* = 0.967) models all indicated good fitting effects. Notably, while the pseudo-second-order model showed the highest mathematical fitting degree, this did not exclusively indicate a chemically controlled process [50]. A critical observation was that the equilibrium adsorption capacity predicted by the pseudo-first-order model (117.314 mg/g) aligned more closely with the experimental value (120.629 mg/g) than that predicted by the pseudo-second-order model. This suggested that the overall kinetics may be more representative of a process linearly dependent on the concentration of available adsorption sites, a characteristic often associated with dominant physical interactions such as micropore filling, van der Waals forces, and π-π interactions [18,51]. Concurrently, the applicability of the Elovich model reflects the heterogeneous surface energy distribution of LPHC. Therefore, the kinetic analysis, combined with the equilibrium isotherm study, indicated that the adsorption of TC onto LPHC was a synergistic process governed by both physical and chemical interactions.

To deeply explore the mass transfer mechanism, the kinetics of the adsorption process was analyzed using the intraparticle diffusion model (Figure 6b and Table 2). The plot of *qₜ* versus *t*^0^·^5^ exhibited three distinct linear regions. The sequence of rate constants (*kᵢ*_1_ > *kᵢ*_2_ > *kᵢ*_3_) indicated a gradual decrease in the mass transfer rate. The *kᵢ*_1_ in the first stage is the largest, corresponding to the rapid migration of TC molecules across the boundary layer to the adsorbent’s external surface. The *kᵢ*_2_ in the second stage decreased significantly, indicating that the pore diffusion within the particle gradually became the rate-determining step. The *kᵢ*_3_ in the third stage was the smallest, reflecting that the mass transfer rate further decreased and approaches the adsorption equilibrium state. Additionally, the non-zero intercepts confirmed that boundary layer resistance exerted a significant influence from the outset and persisted throughout the process [24]. In summary, the kinetic behavior of this adsorption process was not controlled by a single diffusion mechanism but was determined by both boundary layer diffusion and intraparticle diffusion.

### 2.7. Isothermal Adsorption Behavior

**Figure 7:**
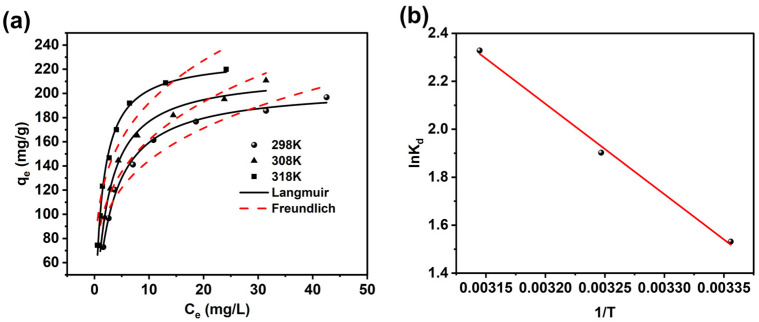
(**a**) The Langmuir model and Freundlich model fitting curves. (**b**) Thermodynamics of the adsorption of TC on LPHC. (Adsorption conditions: *C_0_* = 60–200 mg/L; adsorbent dose = 0.04 g; time = 480 min; *V_0_* = 50 mL, pH = 6).

To clarify the adsorption mechanism of TC on LPHC and its temperature dependence, the adsorption isotherm data were fitted with the Langmuir and Freundlich models at 298 K, 308 K and 318 K in this study. As shown in Figure 7a, the adsorption capacity at each temperature gradually increased and tends to saturation with the increase in equilibrium concentration (*Ce*), with a maximum adsorption capacity of 219.81 mg/g, demonstrated the typical characteristics of monolayer adsorption. Meanwhile, the adsorption isotherms shifted systematically upward with the increase in temperature, indicating that the adsorption capacity was enhanced by raising the temperature. The fitting results (see Table 3) revealed that the correlation coefficients (*R^2^* > 0.99) of the Langmuir model at all temperatures were significantly higher than those of the Freundlich model, suggesting that the former could more accurately describe the adsorption behavior over the entire concentration range. This further confirmed that the adsorption mainly occurred on the surface with relatively uniform energy distribution and formed a monolayer structure. In-depth analysis of the Langmuir parameters revealed the key influence of temperature: the theoretical maximum adsorption capacity (*q_m_*) increased from 205.433 mg/g at 298 K to 214.653 mg/g at 308 K and reached 229.765 mg/g at 318 K; meanwhile, the constant K_L_, which reflects the adsorption affinity, also showed an increasing trend, i.e., *K_L_*, 298 K (0.346 L/mg) < *K_L_*, 308 K (0.453 L/mg) < *K_L_*, 318 K (0.744 L/mg), indicating that the increase in temperature not only enhanced the limit adsorption capacity of the material but also strengthened the binding ability of the adsorption sites to TC molecules. Additionally, the separation factor *R_L_* calculated based on *K_L_* was between 0 and 1 under all experimental conditions, indicating that the adsorption process was a favorable and easily occurring one. In conclusion, the adsorption isotherm analysis demonstrated that the adsorption of TC on LPHC was a spontaneous monolayer adsorption process that conformed to the Langmuir model, and the increase in temperature significantly improved the adsorption performance by synergistically enhancing the adsorption capacity and intermolecular forces.

To explore the thermodynamic characteristics of the adsorption process of TC by LPHC, the standard thermodynamic parameters *ΔH^θ^*, *ΔS^θ^* and *ΔG^θ^* were calculated based on the adsorption equilibrium data at different temperatures using the slope and intercept of the Van’t Hoff plot (Figure 7b). The results are listed in Table 4. As shown in the table, *ΔG^θ^* at 298 K, 308 K and 318 K were −7.42, −9.01 and −10.60 kJ·mol^−1^, respectively, all of which were negative and their absolute values increased with the rising temperature, indicating that the adsorption process was thermodynamically spontaneous and the increase in temperature was conducive to promoting the reaction in the positive direction. *ΔH^θ^* was 39.24 kJ·mol^−1^, which was positive, indicating that the adsorption process is an endothermic reaction. *ΔS^θ^* was 0.156 kJ·mol^−1^·K^−1^, also positive, reflecting an increase in the molecular degrees of freedom at the solid–liquid interface and an increase in the disorder of the system. In conclusion, the thermodynamic analysis showed that the adsorption of TC by LPHC was a spontaneous, endothermic and entropy-increasing process, and the increase in temperature can significantly promote the adsorption reaction.

### 2.8. Study on Cycling Performance

**Figure 8:**
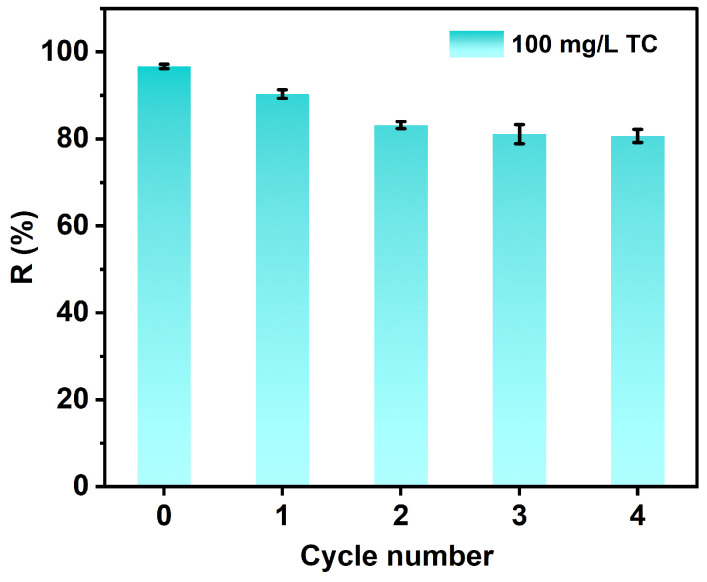
Recyclability of LPHC for adsorption of TC.

Recyclability is key to obtaining economically effective wastewater treatment adsorbents. Therefore, continuous adsorption–desorption cycling processes were employed to evaluate the regeneration capacity of LPHC. After each adsorption saturation, the material was regenerated using a 0.1 M NaOH solution. The alkaline eluent was selected to exploit the competitive interaction between OH^−^ ions and TC molecules and to break possible ionic bonds between TC and acidic functional groups on the carbon surface, thereby achieving efficient desorption. The cycling performance is shown in Figure 8. After four cycles, the TC removal efficiency by LPHC was maintained at 80.27%, indicating good regeneration stability. The slight decrease in removal efficiency was likely attributed to the irreversible adsorption of a small fraction of TC, which may form strong complexes with functional groups on the LPHC surface and could not be desorbed under the given conditions [52].

### 2.9. Possible Adsorption Mechanism

**Figure 9:**
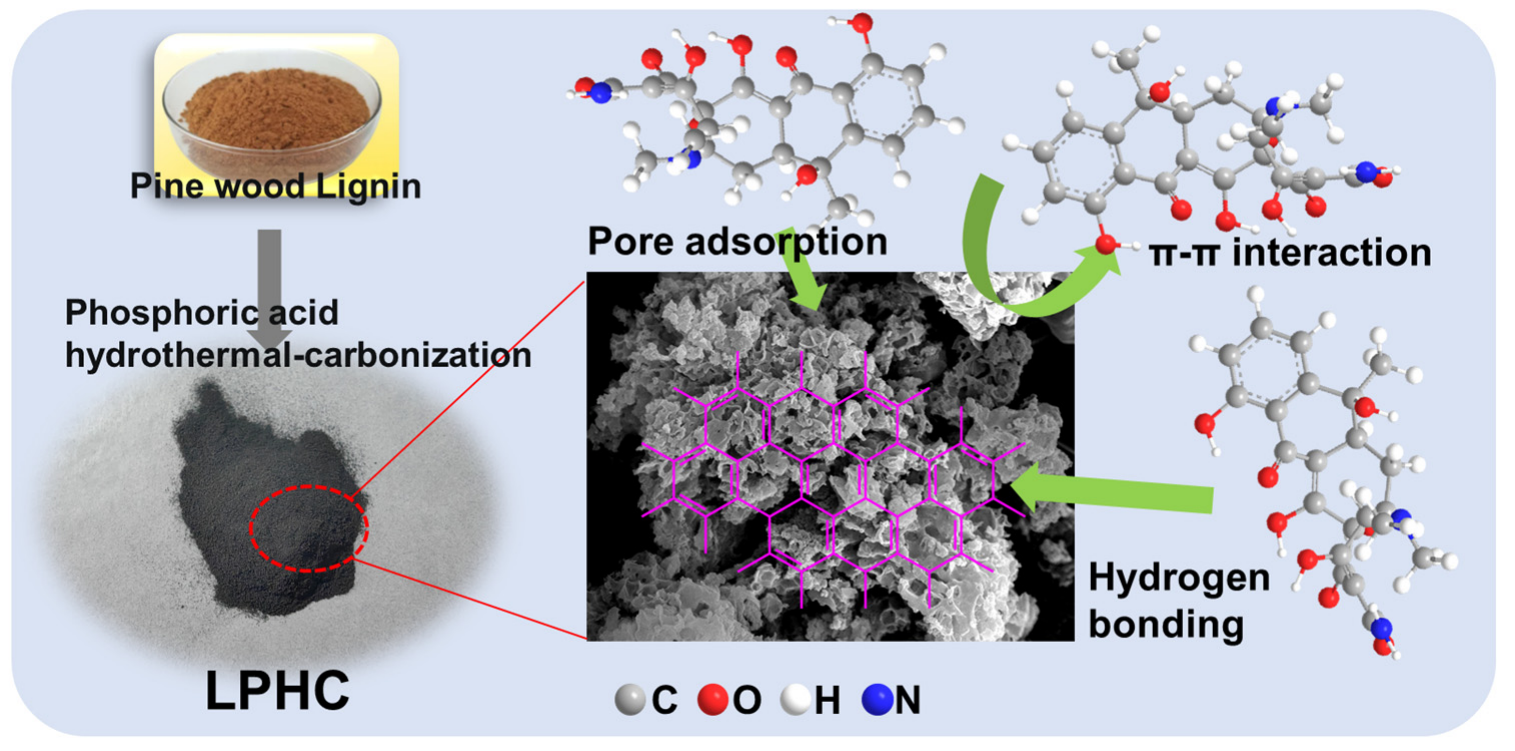
The possible adsorption mechanisms of TC on LPHC.

The adsorption of tetracycline by lignin-derived porous carbon is a process driven by multiple mechanisms working in concert. Its high adsorption efficiency is mainly attributed to the unique multi-level pore structure and rich surface chemical properties of the material. As shown in Figure 9. Firstly, the well-developed pore structure provides a foundation for rapid molecular diffusion and effective pore filling, and the matching between the size of the adsorbate molecules and the pore structure is a key factor determining the adsorption capacity [26,39]. Secondly, the inherent high aromaticity of the lignin carbon skeleton and the oxygen-containing functional groups (C=O, C-O) on its surface can act as hydrogen bond acceptors or π-electron acceptors, forming hydrogen bonds and π-π donor–acceptor interactions with the corresponding groups in TC molecules [6,24,53]. These surface chemical interactions significantly enhance the adsorption selectivity and binding strength. In addition, the pH of the solution regulates the electrostatic attraction or repulsion effect by influencing the surface charge state of the adsorbent and the ionization form of TC. In summary, the adsorption of TC by lignin-derived porous carbon is the result of the synergistic action of multiple physical and chemical mechanisms, including pore filling, hydrogen bonding, π-π interactions, electrostatic interactions, and surface complexation. To further evaluate the material performance, the adsorption performance of the prepared material in this study was compared with various typical biomass derived porous carbons, and the results are summarized in Table 5. Under relatively mild synthesis conditions, porous carbon materials prepared using lignin as a precursor and phosphoric acid activation method exhibit excellent tetracycline adsorption capacity, surpassing most biochar materials reported in the literature [8,26,33,45,54]. This provides a feasible technical path for the high-value utilization of lignin resources and the treatment of antibiotic pollution in water environments.

## 3. Materials and Methods

### 3.1. Materials

Pine Wood Lignin (Average Molecular Weight > 10,000 Da, Content 60–70%, pH 8.0) was purchased from Yanghai Environmental Protection Materials Co., Ltd (Jinan, China). Phosphoric acid (H_3_PO_4_), hydrochloric acid (HCl, 30%) and ethanol (EtOH, AR) were purchased from the Sinopharm Chemical Reagent Co., Ltd (Shanghai, China). Tetracycline (TC) were provided by Macklin Biochemical Co., Ltd (Shanghai, China). All chemicals were used as received without further purification. Deionized water was obtained through a distilled water device in the lab.

### 3.2. Synthesis of Lignin-Derived Hierarchical Porous Carbon

**Scheme 1:**
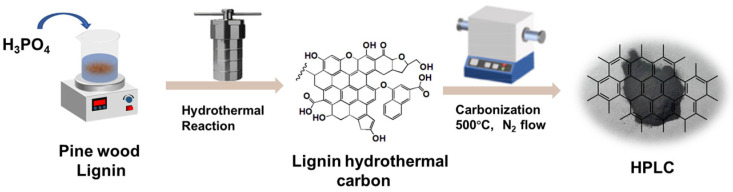
Fabrication process schematic of the LPHC.

Pine wood lignin was mixed with a 50% phosphoric acid solution at a mass ratio of 1.5:1 (phosphoric acid to lignin) to form a homogeneous slurry. The phosphoric acid-impregnated slurry was transferred into an autoclave equipped with a polytetrafluoroethylene liner and hydrothermally treated at 200 °C for 18 h to yielding a coked precursor. The coked sample was dried at 80 °C for 6 h and subjected to a two-stage pyrolysis program in a tube furnace under nitrogen atmosphere. The first stage involved heating to 250 °C at a rate of 1 °C/min and heating for 120 min with a nitrogen flow of 60 mL/min, while the second stage raised the temperature to 500 °C at 3 °C/min and maintained it for 90 min with a nitrogen flow of 200 mL/min. The pyrolyzed product was immersed in 0.1 mol/L HCl solution for 24 h to eliminate phosphate residues, washed with hot water (80 °C) to remove residual impurities, and dried to obtain the lignin hydrothermal–phosphoric acid co-activated carbonized carbon (LPHC). The synthesis flowchart of LPHC in Scheme 1. For comparative, lignin-derived porous carbon (LC) was synthesized through direct carbonization without phosphoric acid activation. Lignin phosphoric acid activated porous carbon (LPC) was prepared by immersing in phosphoric acid at room temperature for 6 h and then carbonizing at 500 °C, with other parameters remaining the same.

### 3.3. Characterization

The surface functional groups of the adsorbent were characterized using Fourier transform infrared spectroscopy (FTIR, Thermo Scientific iS50, Waltham, USA) within a scanning range of 4000–400 cm^−1^. The crystal structure of the samples was analyzed by X-ray diffraction (XRD, Bruker D8 ADVANCE, Karlsruhe, Germany), with data collected over a 2θ range of 10–80° at a scanning rate of 4°/min and a step size of 0.02°. Surface morphology was examined via scanning electron microscopy (SEM, Hitachi SU8010, Tokyo, Japan). The specific surface area and pore structure were determined using a automated surface area and porosity analyzer (V-Sorb-4800P, Beijing, China), with nitrogen adsorption–desorption isotherms recorded at 77 K. Prior to analysis, the samples were degassed under vacuum at 150 °C for 6 h. The chemical states and elemental composition of the sample surfaces were investigated by X-ray photoelectron spectroscopy (XPS; Thermo Scientific ESCALAB 250Xi, USA), with binding energies referenced to the C 1s peak at 284.8 eV. The zeta potential of the adsorbent under different pH conditions was determined by using a potential analyzer (Zetatronix 939SZ, Shanghai, China).

### 3.4. Batch Adsorption Experiments

Three as-prepared porous carbon materials were used for TC solution adsorption experiments. The detailed procedure was as follows: 0.04 g of the adsorbent was placed in 50 mL of TC solution with an initial concentration of 100 mg/L, and the mixture was shaken in a constant temperature air bath shaker at 25 °C and 150 r/min. Samples were taken at set time points within 0 to 480 min, filtered through a 0.22 μm filter membrane, and the residual TC concentration in the filtrate was determined at a wavelength of 357 nm using a UV-visible spectrophotometer. LPHC was selected as the target adsorbent to conduct further optimization experiments of adsorption conditions. The effects of adsorbent dosage and initial TC concentration on the adsorption performance were investigated, respectively. The influence of solution pH was explored by adjusting the pH to the range of 2–10 using 0.01 mol/L NaOH or 0.01 mol/L HCl. The adsorption performance was calculated by adsorption capacity *q_e_* (mg/g) and removal efficiency *R* (%), which were calculated by Formulas (1) and (2). All experiments were conducted in triplicate, with results reported as the mean of three replicates.

(1) $$q_{e}=\frac{\left(C_{0}-C_{t}\right)}{m}\times V$$

(2) $$R\;(\%)=\frac{\left(C_{0}-C_{t}\right)}{C_{0}}\times 100\%$$

where *C*_0_ (mg/L) and *C_t_* (mg/L) represent the initial and time-dependent tetracycline concentrations, respectively; *V* (mL) is the solution volume; and *m* (g) denotes the mass of adsorbent.

In the adsorption kinetics experiment, 0.04 g of LPHC was added to 50 mL of TC solution with an initial concentration of 100 mg/L. The mixture was stirred magnetically at 298 K and 150 rpm. Samples were taken at preset time points (0–480 min) to determine the concentration changes in TC in the solution. The adsorption isotherm experiments were conducted under the initial TC concentration range of 60–200 mg/L. The reactions were oscillated at 298, 308, and 318 K for 480 min to reach adsorption equilibrium, and then samples were taken and the residual concentration of TC in the filtrate was determined. All experiments were independently repeated three times, and the results were expressed as the average values.

The adsorption process was modeled and analyzed using both linear and nonlinear fitting methods. The kinetic behavior was fitted by pseudo-first-order, pseudo-second-order and Elovich kinetic equations, and the possible diffusion mechanisms were explored in combination with the intraparticle diffusion model. The adsorption isotherm behavior was described by Langmuir and Freundlich models. The specific expressions and parameters of each model are detailed in the Supplementary Materials. Data processing and curve fitting were all accomplished using Origin 2024 software.

### 3.5. The Adsorption and Regeneration Performance of LPHC

To evaluate the reusability of LPHC, five consecutive adsorption–desorption cycles were conducted. In each cycle, adsorption was performed under the optimized conditions (0.8 g/L LPHC, 100 mg/L TC, pH=6, 25 °C, 480 min). After saturation, the material was separated by centrifugation, rinsed three times with deionized water, and then desorbed in 0.1 mol/L NaOH solution at 30 °C and 150 rpm for 12 h. The desorbed material was washed sequentially with deionized water and anhydrous ethanol, dried at 105 °C to constant weight, and reused in the next cycle with fresh TC solution. This procedure was repeated five times to assess the performance stability of the regenerated LPHC.

## 4. Conclusions

In this study, a hierarchical porous carbon material based on lignin was successfully prepared through a phosphoric acid-assisted hydrothermal carbonization coupling process. This process effectively constructed a high specific surface area and well-developed mesoporous structure, providing abundant active sites and efficient mass transfer channels for the adsorption of tetracycline. When the initial TC concentration was 100 mg·L^−1^, the adsorbent dosage was 0.04 g, and the solution pH was 6, the maximum adsorption capacity of LPHC for TC reached 120.63 mg·g^−1^, with a removal rate as high as 96.33%, which was significantly superior to that of directly carbonized or only phosphoric acid-activated porous carbon materials. The adsorption process conforms to the Langmuir isotherm adsorption model, indicating that adsorption mainly occurs in the form of surface monolayer adsorption, with a maximum adsorption capacity of 219.81 mg·g^−1^ at 318 k. Mechanism analysis revealed that the adsorption process was mainly driven by multiple synergistic mechanisms, including pore filling, hydrogen bonding, π-π donor–acceptor interactions, and electrostatic attraction. This study provides a practical and feasible technical approach for the high-value utilization of lignin resources and the treatment of antibiotic-contaminated water.

## Figures and Tables

**Table 1:** Pore structure parameters of LC, LPC and LPHC.

Samples	Specific Surface Area (m^2^/g)	Pore Volume (cm^3^/g)	Average Pore Size (nm)
LPHC	1157.25	0.60	2.12
LPC	720.11	0.55	2.48
LC	29.61	0.051	6.92

**Table 2:** Adsorption kinetic parameters for TC on LPHC.

Models	Parameters	LPHC
Pseudo-first-order (PFO)	*K_1_* (min^−1^)	0.0275
	*q_e_, cal* (mg g^−1^)	117.314
	*R^2^*	0.987
Pseudo-second-order (PSO)	*K_2_* (g mg^−1^ min^−1^)	2.712
	*q_e_, cal* (mg g^−1^)	130.930
	*R^2^*	0.995
Elovich	*α* (mg g^−1^ min^−1^)	12.085
	*β* (g mg^−1^)	0.0415
	*R^2^*	0.967
Intraparticle diffusion (IPD)	*k_i1_*	14.640
	*C_1_*	−13.203
	*R^2^*	0.947
	*k_i2_*	5.245
	*C_2_*	47.061
	*R^2^*	0.942
	*k_i3_*	0.658
	*C_3_*	107.421
	*R^2^*	0.655

**Table 3:** Isotherm model parameters of TC adsorption on LPHC.

*T* (K)	Langmuir Model			Freundlich Model		
	*K_L_* (L mg^−1^)	*q_m_* (mg g^−1^)	*R^2^*	*K_F_* (L mg^−1^)	*1/n*	*R^2^*
298	0.346	205.433	0.993	82.034	0.331	0.919
308	0.453	214.653	0.994	90.734	0.323	0.928
318	0.744	229.765	0.991	110.014	0.297	0.903

**Table 4:** Adsorption Thermodynamic Parameters for TC Adsorption on LPHC.

*T* (K)	*∆G^θ^* (kJ mol^−1^)	*∆H^θ^* (kJ mol^−1^)	*∆S^θ^* (kJ mol^−1^ K^−1^)
298	−7.42	39.24	0.156
308	−9.01		
318	−10.60		

**Table 5:** Comparison of TC adsorption capacity of different biomass-derived carbon materials.

Biomass	Method	Carbonization Temperature (°C)	*q_m_* (mg/g)	Ref
Copper pod tree leaves	phosphoric acid-activated	400	103.32	[8]
Typha	KOH and ZnCl_2_-activated	800	134.48	[33]
Grape leaves	Direct carbonization	900	52.8	[45]
residue of Flueggea suffruticosa	ZnCl_2_-activated	500	188.7	[26]
Bagasse	ZnAl-layered double hydroxide modified	600	41.98	[54]
Pine wood Lignin	Phosphoric acid hydrothermal assisted pyrolysis	500	219.81	This work

## Data Availability

The data presented in this study are available on request from the corresponding author.

## Supplementary Materials

The following supporting information can be downloaded at https://www.mdpi.com/article/10.3390/molecules31030447/s1: Adsorption kinetic equation, Adsorption isotherm equation, Thermodynamic equation and Figure S1: The XPS full spectrum of LPHC and LPC.
