# Supplementary material for: Synthesis of Lignin-Derived Hierarchical Porous Carbon via Hydrothermal–Phosphoric Acid Synergistic Activation for Enhanced Adsorption of Tetracycline
Source: Molecules. 2026 Jan 27;31(3):447. doi: 10.3390/molecules31030447 (PMC12899767; doi:10.3390/molecules31030447)
Supplement: Supplementary file 1 [file molecules-31-00447-s001.zip › molecules-4084370-supplementary.pdf]

Supporting Information

Synthesis of Lignin-Derived Hierarchical Porous Carbon via Hydrothermal-Phosphoric Acid Synergistic Activation for Enhanced Adsorption of Tetracycline

Xin Li[1,*], Yipeng Li[1], Yuhan Li[1], Mengyu Zhang[2], Jundong Zhu [2,*]

1. Hunan Provincial Key Laboratory of the Research and Development of Novel Pharmaceutical Preparations, College of Pharmacy, Changsha Medical University, Hunan, 410219, China

2. Hunan Provincial Key Laboratory of Carbon Neutrality and Intelligent Energy, School of Resources and Environment, Hunan University of Technology and Business, Hunan, 410205, China

*Corresponding author:

tengyunxin2010@163.com (X. Li)

zhujundong@hutb.edu.cn (JD. Zhu)

Adsorption kinetic equation

The following Eqs. (S1)-(S4) were used to fit the adsorption kinetic data, respectively:

Pseudo-first-order:

$$ln(q_e - q_t) = lnq_e - k_1 t \tag{S1}$$

Pseudo-second-order:

$$\frac{t}{q_t} = \frac{1}{k_2 {q_e}^2} + \frac{t}{q_e} \tag{S2}$$

Elovich:

$$q_t = \beta \ln(\alpha\beta) + \ln(t) \tag{S3}$$

and Intra-particle diffusion model:

$$q_t = k_t t^{0.5} + C \tag{S4}$$

where q_e ($mg \cdot g^{-1}$) is the adsorbed amount of tetracycline at adsorption equilibrium; q_t ($mg \cdot g^{-1}$) is the adsorbed amount of tetracycline at time *t*; k_1 (min^{-1}), k_2 ($g \cdot mg^{-1} \cdot min^{-1}$) are PFO and *PSO* adsorption rate constants; α ($mg \cdot g^{-1} \cdot min^{-1}$) is the beginning adsorption rate constants and β ($g \cdot mg^{-1}$) is the desorption rate constants, and *C* is the intercept of the intra-particle diffusion model.

Adsorption isotherm equation

The following Eqs. (S5)-(S6) were used to fit the adsorption isotherm data, respectively:

Langmuir model:

$$q_e = \frac{q_m K_L C_e}{1 + K_L C_e} \tag{S5}$$

Freundlich model:

$$q_e = K_F {C_e}^{1/n} \tag{S6}$$

Where q_m ($mg \cdot g^{-1}$) is the theoretical maximum adsorption capacity; C_e ($mg \cdot L^{-1}$) is the

concentration of solution at adsorption equilibrium; K_L ($L \cdot mg^{-1}$), K_F ($mg \cdot g^{-1} \cdot (L \cdot mg^{-1})^{1/n}$) and n are Langmuir and Freundlich constants, respectively.

Thermodynamic equation

The thermodynamic equations are presented in Eqs. (S7)-(S9);

$$K_d = \frac{q_e}{C_e} \tag{S7}$$

$$ln\, K_d = -\frac{\Delta H}{RT} + \frac{\Delta S}{R} \tag{S8}$$

$$\Delta G = -RT\, ln\, K_d \tag{S9}$$

Where ΔG (kJ/mol) is the Gibbs free energy; ΔH ($kJ \cdot mol^{-1}$) is the enthalpy change; ΔS ($J \cdot mol^{-1} \cdot K^{-1}$) is the entropy change; R (8.314 $J \cdot mol^{-1} \cdot K^{-1}$) is gas constant; T (K) is the temperature, K_d is the thermodynamic equilibrium constant.

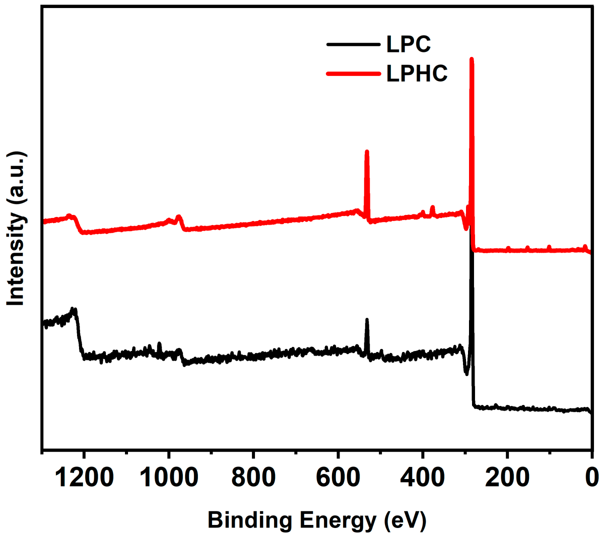

Figure S1. The XPS full spectrum of LPC and LPHC
